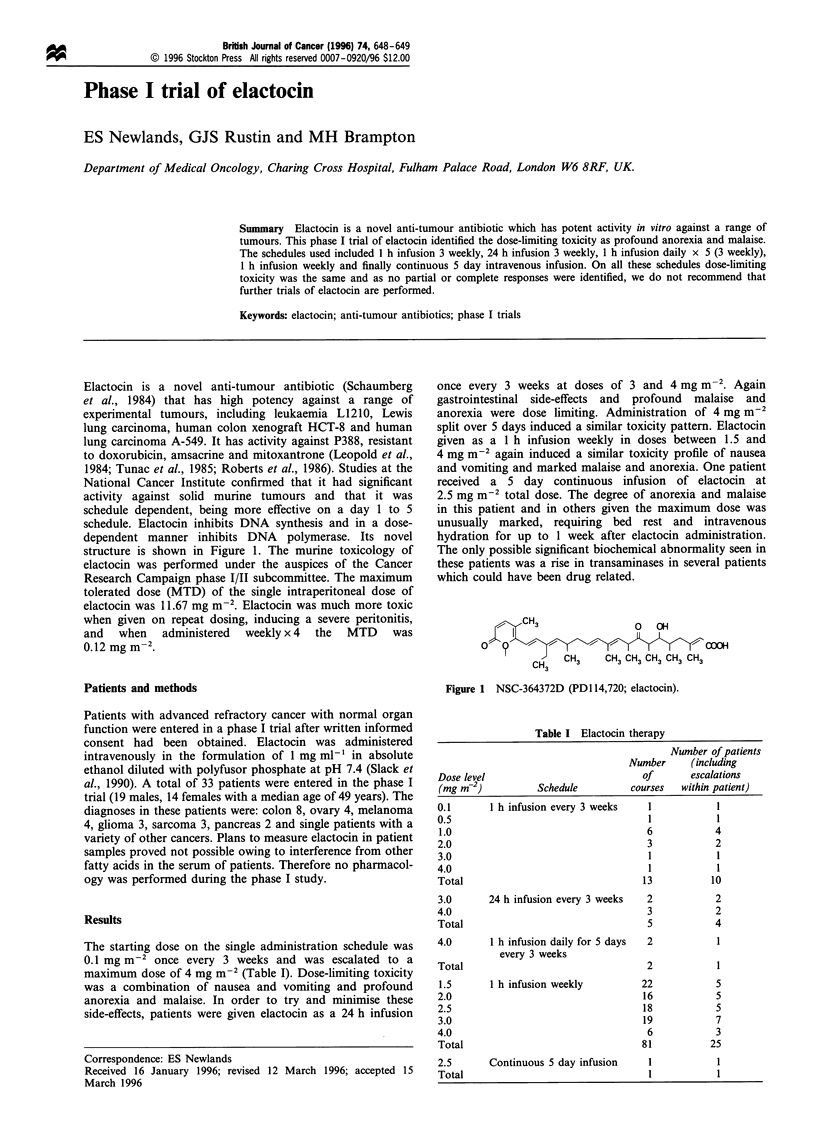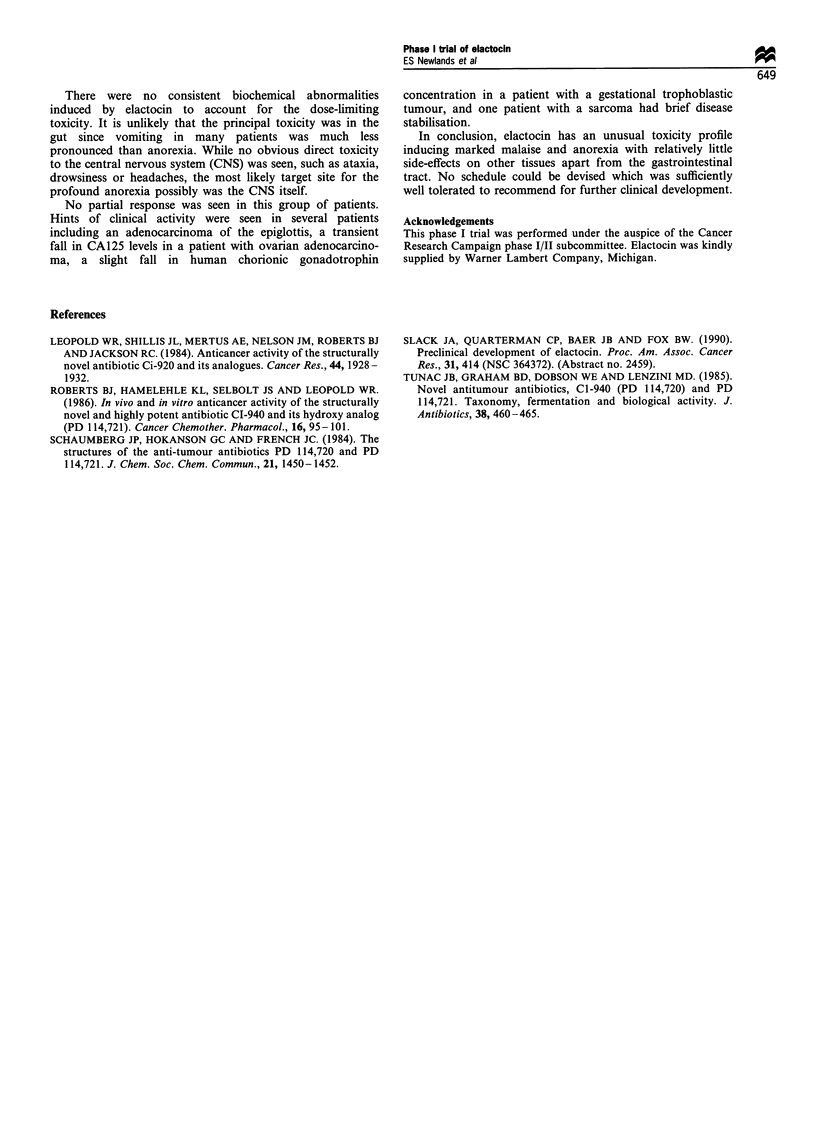# Phase I trial of elactocin.

**DOI:** 10.1038/bjc.1996.415

**Published:** 1996-08

**Authors:** E. S. Newlands, G. J. Rustin, M. H. Brampton

**Affiliations:** Department of Medical Oncology, Charing Cross Hospital, London, UK.

## Abstract

Elactocin is a novel anti-tumour antibiotic which has potent activity in vitro against a range of tumours. This phase I trial of elactocin identified the dose-limiting toxicity as profound anorexia and malaise. The schedules used included 1 h infusion 3 weekly, 24 h infusion 3 weekly, 1 h infusion daily x 5 (3 weekly), 1 h infusion weekly and finally continuous 5 day intravenous infusion. On all these schedules dose-limiting toxicity was the same and as no partial or complete responses were identified, we do not recommend that further trials of elactocin are performed.


British Journal of Cancer (1996) 74, 648-649
© 1996 Stockton Press All rights reserved 0007-0920/96 $12.00

### Phase I trial of electocin

ES Newlands, GJS Rustin and MH Brampton

*Department of Medical Oncology, Charing Cross Hospital, Fulham Palace Road, London W6 8RF, UK.*

**Summary** Elactocin is a novel anti-tumour antibiotic which has potent activity *in vitro* against a range of tumours. This phase I trial of elactocin identified the dose-limiting toxicity as profound anorexia and malaise. The schedules used included 1 h infusion 3 weekly, 24 h infusion 3 weekly, 1 h infusion daily × 5 (3 weekly), 1 h infusion weekly and finally continuous 5 day intravenous infusion. On all these schedules dose-limiting toxicity was the same and as no partial or complete responses were identified, we do not recommend that further trials of elactocin are performed.

**Keywords:** elactocin; anti-tumour antibiotics; phase I trials

Elactocin is a novel anti-tumour antibiotic (Schaumberg *et al.*, 1984) that has high potency against a range of experimental tumours, including leukaemia L1210, Lewis lung carcinoma, human colon xenograft HCT-8 and human lung carcinoma A-549. It has activity against P388, resistant to doxorubicin, amsacrine and mitoxantrone (Leopold *et al.*, 1984; Tunac *et al.*, 1985; Roberts *et al.*, 1986). Studies at the National Cancer Institute confirmed that it had significant activity against solid murine tumours and that it was schedule dependent, being more effective on a day 1 to 5 schedule. Elactocin inhibits DNA synthesis and in a dose-dependent manner inhibits DNA polymerase. Its novel structure is shown in Figure 1. The murine toxicology of elactocin was performed under the auspices of the Cancer Research Campaign phase I/II subcommittee. The maximum tolerated dose (MTD) of the single intraperitoneal dose of elactocin was 11.67 mg $m^{-2}$. Elactocin was much more toxic when given on repeat dosing, inducing a severe peritonitis, and when administered weekly × 4 the MTD was 0.12 mg $m^{-2}$.

### Patients and methods

Patients with advanced refractory cancer with normal organ function were entered in a phase I trial after written informed consent had been obtained. Elactocin was administered intravenously in the formulation of 1 mg $ml^{-1}$ in absolute ethanol diluted with polyfusor phosphate at pH 7.4 (Slack *et al.*, 1990). A total of 33 patients were entered in the phase I trial (19 males, 14 females with a median age of 49 years). The diagnoses in these patients were: colon 8, ovary 4, melanoma 4, glioma 3, sarcoma 3, pancreas 2 and single patients with a variety of other cancers. Plans to measure elactocin in patient samples proved not possible owing to interference from other fatty acids in the serum of patients. Therefore no pharmacology was performed during the phase I study.

### Results

The starting dose on the single administration schedule was 0.1 mg $m^{-2}$ once every 3 weeks and was escalated to a maximum dose of 4 mg $m^{-2}$ (Table I). Dose-limiting toxicity was a combination of nausea and vomiting and profound anorexia and malaise. In order to try and minimise these side-effects, patients were given elactocin as a 24 h infusion once every 3 weeks at doses of 3 and 4 mg $m^{-2}$. Again gastrointestinal side-effects and profound malaise and anorexia were dose limiting. Administration of 4 mg $m^{-2}$ split over 5 days induced a similar toxicity pattern. Elactocin given as a 1 h infusion weekly in doses between 1.5 and 4 mg $m^{-2}$ again induced a similar toxicity profile of nausea and vomiting and marked malaise and anorexia. One patient received a 5 day continuous infusion of elactocin at 2.5 mg $m^{-2}$ total dose. The degree of anorexia and malaise in this patient and in others given the maximum dose was unusually marked, requiring bed rest and intravenous hydration for up to 1 week after elactocin administration. The only possible significant biochemical abnormality seen in these patients was a rise in transaminases in several patients which could have been drug related.

**Figure 1** NSC-364372D (PD114,720; elactocin).

**Table I** Elactocin therapy

| *Dose level (mg $m^{-2}$)* | *Schedule* | *Number of courses* | *Number of patients (including escalations within patient)* |
|---|---|---|---|
| 0.1 | 1 h infusion every 3 weeks | 1 | 1 |
| 0.5 | | 1 | 1 |
| 1.0 | | 6 | 4 |
| 2.0 | | 3 | 2 |
| 3.0 | | 1 | 1 |
| 4.0 | | 1 | 1 |
| Total | | 13 | 10 |
| 3.0 | 24 h infusion every 3 weeks | 2 | 2 |
| 4.0 | | 3 | 2 |
| Total | | 5 | 4 |
| 4.0 | 1 h infusion daily for 5 days every 3 weeks | 2 | 1 |
| Total | | 2 | 1 |
| 1.5 | 1 h infusion weekly | 22 | 5 |
| 2.0 | | 16 | 5 |
| 2.5 | | 18 | 5 |
| 3.0 | | 19 | 7 |
| 4.0 | | 6 | 3 |
| Total | | 81 | 25 |
| 2.5 | Continuous 5 day infusion | 1 | 1 |
| Total | | 1 | 1 |

Correspondence: ES Newlands
Received 16 January 1996; revised 12 March 1996; accepted 15 March 1996

There were no consistent biochemical abnormalities induced by elactocin to account for the dose-limiting toxicity. It is unlikely that the principal toxicity was in the gut since vomiting in many patients was much less pronounced than anorexia. While no obvious direct toxicity to the central nervous system (CNS) was seen, such as ataxia, drowsiness or headaches, the most likely target site for the profound anorexia possibly was the CNS itself.

No partial response was seen in this group of patients. Hints of clinical activity were seen in several patients including an adenocarcinoma of the epiglottis, a transient fall in CA125 levels in a patient with ovarian adenocarcinoma, a slight fall in human chorionic gonadotrophin concentration in a patient with a gestational trophoblastic tumour, and one patient with a sarcoma had brief disease stabilisation.

In conclusion, elactocin has an unusual toxicity profile inducing marked malaise and anorexia with relatively little side-effects on other tissues apart from the gastrointestinal tract. No schedule could be devised which was sufficiently well tolerated to recommend for further clinical development.

**Acknowledgements**
This phase I trial was performed under the auspice of the Cancer Research Campaign phase I/II subcommittee. Elactocin was kindly supplied by Warner Lambert Company, Michigan.

### References

LEOPOLD WR, SHILLIS JL, MERTUS AE, NELSON JM, ROBERTS BJ AND JACKSON RC. (1984). Anticancer activity of the structurally novel antibiotic Ci-920 and its analogues. *Cancer Res.*, **44**, 1928–1932.

ROBERTS BJ, HAMELEHLE KL, SELBOLT JS AND LEOPOLD WR. (1986). *In vivo* and *in vitro* anticancer activity of the structurally novel and highly potent antibiotic CI-940 and its hydroxy analog (PD 114,721). *Cancer Chemother. Pharmacol.*, **16,** 95–101.

SCHAUMBERG JP, HOKANSON GC AND FRENCH JC. (1984). The structures of the anti-tumour antibiotics PD 114,720 and PD 114,721. *J. Chem. Soc. Chem. Commun.*, **21,** 1450–1452.

SLACK JA, QUARTERMAN CP, BAER JB AND FOX BW. (1990). Preclinical development of elactocin. *Proc. Am. Assoc. Cancer Res.*, **31,** 414 (NSC 364372). (Abstract no. 2459).

TUNAC JB, GRAHAM BD, DOBSON WE AND LENZINI MD. (1985). Novel antitumour antibiotics, C1-940 (PD 114,720) and PD 114,721. Taxonomy, fermentation and biological activity. *J. Antibiotics*, **38,** 460–465.